# Multimodal imaging study of the 5-HT_1A_ receptor biased agonist, NLX-112, in a model of L-DOPA-induced dyskinesia

**DOI:** 10.1016/j.nicl.2023.103497

**Published:** 2023-08-15

**Authors:** Sarah Chaib, Benjamin Vidal, Caroline Bouillot, Ronan Depoortere, Adrian Newman-Tancredi, Luc Zimmer, Elise Levigoureux

**Affiliations:** a Université Claude Bernard Lyon 1, Lyon Neuroscience Research Center, CNRS, INSERM, Lyon, France; b Hospices Civils de Lyon, Lyon, France; c CERMEP-Imaging Platform, Bron, France; d Neurolixis, Castres, France

**Keywords:** FDG, fMRI, Serotonin, Levodopa-induced dyskinesia, Biased agonist

## Abstract

**Introduction:**

The leading treatment for motor signs of Parkinson's disease is L-DOPA, but, upon extended use, it can lead to levodopa-induced dyskinesia (LID). Serotonergic neurons are involved in LID etiology and previous pre-clinical studies have shown that NLX-112, a 5-HT_1A_ biased agonist, has robust antidyskinetic effects. Here, we investigated its effects in hemiparkinsonian (HPK) rats with a unilateral nigrostriatal 6-OHDA lesion.

**Methods:**

We compared HPK rats with LID (i.e., sensitized to the dyskinetic effects of chronic L-DOPA) and without LID (HPK-non-LID), using [^18^F]FDG PET imaging and fMRI functional connectivity following systemic treatment with saline, L-DOPA, NLX-112 or L-DOPA + NLX-112.

**Results:**

In HPK-non-LID rats, [^18^F]FDG PET experiments showed that L-DOPA led to hypermetabolism in motor areas (cerebellum, brainstem, and mesencephalic locomotor region) and to hypometabolism in cortical regions. L-DOPA effects were also observed in HPK-LID rats, with the additional emergence of hypermetabolism in raphe nuclei and hypometabolism in hippocampus and striatum. NLX-112 attenuated L-DOPA-induced raphe hypermetabolism and cingulate cortex hypometabolism in HPK-LID rats. Moreover, in fMRI experiments NLX-112 partially corrected the altered neural circuit connectivity profile in HPK-LID rats, through activity in regions rich in 5-HT_1A_ receptors.

**Conclusion:**

This neuroimaging study sheds light for the first time on the brain activation patterns of HPK-LID rats. The 5-HT_1A_ receptor agonist, NLX-112, prevents occurrence of LID, likely by activating pre-synaptic autoreceptors in the raphe nuclei, resulting in a partial restoration of brain metabolic and connectivity profiles. In addition, NLX-112 also rescues L-DOPA-induced deficits in cortical activation, suggesting potential benefit against non-motor symptoms of Parkinson’s disease.

## Highlights

• Hemiparkinsonian (HPK) rats show a specific L-DOPA-induced brain metabolic pattern.
• NLX-112 attenuated or reversed the cerebral effects of L-DOPA in dyskinetic HPK rats.
• The predominant action of NLX-112 is mediated by 5-HT1A autoreceptors in the raphe.
• This translational study supports development of NLX-112 as an anti-dyskinetic agent.

## Graphical abstract

**Figure:**
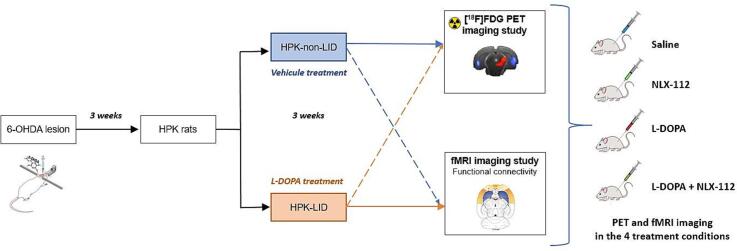

## 1 Introduction

Parkinson’s disease (PD) is the second most common neurodegenerative disorder after Alzheimer’s disease (de Lau and Breteler, 2006); (Lees et al., 2009). The gold standard treatment for PD is the oral administration of the dopamine (DA) precursor, L-3,4-dihydroxyphenylalanine (L-DOPA), which compensates for the progressive reduction of DA release in the projection areas (especially the striatum) (Poewe et al., 2017) due to loss of DAergic neurons in the substantia nigra *pars compacta* (SNc).

Although L-DOPA is an effective treatment to alleviate motor symptoms, its therapeutic efficacy is gradually lost and its long-term use often leads to development of troublesome abnormal involuntary movements (AIMs) also called L-DOPA-induced dyskinesia (LID) (Lees et al., 1977, Marsden and Parkes, 1977). Around 80% of patients will be affected by LID after 10 years of treatment (Grandas et al., 1999). The management of these side-effects related to L-DOPA protracted therapy is problematic and there is no fully efficacious and well tolerated treatment.

While the pathophysiology of LID is still incompletely understood, altered signaling by DA D_1_ receptors (D_1_R) (Bastide et al., 2015), NMDA and AMPA (Bastide et al., 2015) receptors, and striatal serotonergic 5-HT_1A_ receptors (5-HT_1A_R) (Bishop et al., 2009) are probably involved. In particular, the DAergic nigrostriatal denervation seen in PD results in striatal serotonergic neurons taking up and converting L-DOPA into DA through their enzymatic machinery (L-amino acid decarboxylase). Consequently, DA is aberrantly released as a ‘false neurotransmitter’ by serotonergic neurons without the autoregulatory feedback mechanisms specific to DAergic neurons (Carta and Bezard, 2011, Cenci, 2014). In this context, targeting inhibitory serotonergic autoreceptors, such as 5-HT_1A_R located on raphe nuclei, appears to be a promising strategy to reduce swings in synaptic levels of DA, and consequently decrease LID (Huot et al., 2017, Ohno et al., 2013).

It was shown that serotonergic drugs reducing the release of DA by 5-HT terminals in the striatum are able to attenuate LID in several human studies (Politis et al., 2014). Moreover, in non-human primates rendered parkinsonian with the neurotoxin 1-methyl-4-phenyl-1,2,3,6-tetrahydropyridine (MPTP) (Bezard et al., 2013, Rylander et al., 2010), or in hemiparkinsonian rats with a unilateral 6-hydroxydopamine (6-OHDA) (Carta et al., 2007, Farajdokht et al., 2020, Rylander et al., 2010) lesion of the nigrostriatal DA system (HPK rats), appearance of AIMs is dependent on the integrity of serotonergic projections. Thus, AIMs were reduced by removing striatal serotonin afferents (Carta et al., 2007) or by systemic administration of 5-HT_1A_ agonists, e.g. buspirone, sarizotan or eltoprazine (Bezard et al., 2013, Eskow et al., 2007, Muñoz et al., 2009, Muñoz et al., 2008, Pagano and Politis, 2018, Politis et al., 2014).

However, these compounds do not act only on 5-HT_1A_R, but they also, for buspirone and sarizotan, block DAergic receptors (Bartoszyk et al., 2004, Gerlach et al., 2011, Grégoire et al., 2009, Huot, 2018, Kuzhikandathil and Bartoszyk, 2006), which likely interferes with the therapeutic activity of L-DOPA. Furthermore, buspirone and eltoprazine do not fully activate 5-HT_1A_R, and only act as partial agonists (Newman-Tancredi et al., 2021). Thus, there is a place for high efficacy and selective agonists, targeting exclusively 5-HT_1A_R. For this purpose, the novel 5-HT_1A_R biased agonist, NLX-112 (also known as F13640 or befiradol), presents promising antidyskinetic effects in animal models (Depoortere et al., 2020, Fisher et al., 2020, Iderberg et al., 2015, McCreary et al., 2016). NLX-112 recently underwent a successful proof-of-concept clinical trial as a treatment for LID (ClinicalTrials.gov identifier # NCT05148884). In contrast to other 5-HT_1A_R agonists, NLX-112 has the advantage of possessing excellent selectivity and nanomolar affinity at 5-HT_1A_R, and high agonist ‘full efficacy’ at these receptors (Colpaert et al., 2002, Newman-Tancredi et al., 2017; Lladó-Pelfort et al., 2012, Newman-Tancredi et al., 2021, Vidal et al., 2018).

Initial [^18^F]FDG PET imaging studies in naïve rats showed that NLX-112 modifies glucose metabolism in the cerebellum, striatum, brainstem and raphe nuclei (Levigoureux et al., 2019). In fMRI experiments, NLX-112 also elicited functional connectivity effects in regions expressing pre-synaptic 5-HT_1A_R (Vidal et al., 2020). Changes in the functional state of 5-HT_1A_R were also observed in HPK rats, especially when treated repeatedly with L-DOPA to elicit dyskinesia (Vidal et al., 2021).

The aim of this study was to define for the first time the brain activation and functional connectivity profiles in rats presenting LID, based respectively on glucose metabolism changes ([^18^F]FDG PET) and temporal correlations of BOLD signal fluctuations (fMRI imaging), and to visualize the different neural circuits affected by NLX-112 in HPK rats with LID (HPK-LID) or without LID (HPK-non-LID rats).

## 2 Methods

### 2.1 Animals

Seventy-nine adult male Sprague-Dawley rats (Charles River laboratories, France) weighing 200–250 g were used in the present study. Animals were initially housed six per cage in standard temperature (22 ± 2 °C) and humidity (50%) conditions with a 12 h/12 h light/dark cycle, light on at 8.00 am; food and water were provided *ad libitum*. All experiments were performed in accordance with the European guidelines for care of laboratory animals (directive 2010/63/EU) and approved by the University of Lyon review board (CELYNE).

### 2.2 Unilateral 6-OHDA lesions in the medial forebrain bundle

Prior to surgery, buprenorphine (0.05 mg/kg, s.c.) and lidocaine cream were used for analgesia. Anesthesia induction was performed with a mixture of 4% isoflurane and air (1 L/min), lowered to 2% isoflurane during the stereotaxic surgical procedure. Rats were lesioned in the right medial forebrain bundle (MFB) by infusing 6-OHDA (14 µg/4 µL, 1 µL/min) dissolved in saline containing ascorbic acid (0.02%), using the following coordinates from the Paxinos and Watson’s (Iderberg et al., 2015, Paxinos and Watson, 2014) atlas: AP =  − 4.0 mm and ML =  − 1.3 mm, relative to bregma, and DV = -7.6 mm from the dural surface. Following the procedure, rats were single-housed for 24 h, before being housed 6 per cage again for the rest of the experiment. After 3 weeks, rats were challenged with apomorphine (0.05 mg/kg, s.c.) and turning behavior assessed over 30 min post-injection, in order to verify a striatal DAergic depletion of at least 90% (Hudson et al., 1993). Thirty-one rats were selected and used in subsequent neuroimaging experiments and AIMS scoring for 15 of them (see supplementary figure for details).

### 2.3 Autoradiography

After selection with the apomorphine test, an additional cohort of HPK rats (n = 7) was used for validation of the unilateral dopaminergic lesion by autoradiography of the dopamine transporter (DAT). After a short inhalation of isoflurane, rats were decapitated and brains were carefully removed and immediately frozen in 2-methylbutane cooled with dry ice (−29 °C). Coronal sections (30 μm thick) were cut using a − 20 °C cryostat, thaw-mounted on glass slides, and allowed to air dry before storage at − 80 °C until used. The day of [^11^C]PE2I synthesis, the slides were incubated for 20 min in Tris phosphate- buffered saline buffer, pH adjusted to 7.5, containing 37 kBq/ml of [^11^C]PE2I. Non-specific binding was determined in duplicate serial sections co-incubated with GBR12909 10 µM (Sigma-Aldrich). After incubation, the slides were dipped in cold buffer and distilled water (4 °C) and then dried and juxtaposed to a phosphor imaging plate for 120 min (BAS-1800 II; Fujifilm). The striatum was drawn manually using Multigauge software (Fujifilm). The specific binding was determined from the subtraction of the non-specific binding from the total binding in the duplicate sections, and results were expressed in optical densities (PSL/mm^2^).

### 2.4 L-DOPA-induced dyskinesia

Rats of the HPK-LID cohort received chronic daily treatment with a combination of L-DOPA and benserazide (6/12 mg/kg s.c., administered as a single injection) for 3 weeks to induce stable reproducible AIMs.

### 2.5 AIMs ratings following an acute challenge with L-DOPA alone or in combination with NLX-112

Before PET acquisitions, 15 animals (8 HPK-LID rats and 7 HPK-non-LID rats) were video-recorded in order to quantify AIMs following the 4 different *i.p.* treatments, *i.e.*, saline (control condition), L-DOPA/benserazide (6/12 mg/kg), NLX-112 alone (0.16 mg/kg, a fully antidyskinetic dose (Iderberg et al., 2015)) or L-DOPA/benserazide (6/12 mg/kg) + NLX-112 (0.16 mg/kg). AIMs rating sessions began 30 min after treatment and lasted for 5 min. The evaluation of AIMs was adapted from the Cenci and Lundblad protocol (Cenci and Lundblad, 2007) and was scored off-line according to the following scale: 0 = Absence of dyskinesia (normal); 1 = Orolingual dyskinesia; 2 = Orolingual + moderate limb & axial dyskinesia; 3 = Orolingual + pronounced limb & axial dyskinesia. Data were analyzed with a non-parametric Kruskal-Wallis test, followed by a Dunn’s post-hoc multiple comparison test.

### 2.6 PET imaging procedures and data analyses

#### 2.6.1 PET/CT protocol and image acquisition

All 15 rats that were tested in the AIMs scoring experiment were scanned 3 weeks after the apomorphine-induced rotational test. Each animal underwent 4 different PET scans with [^18^F]FDG in a randomized order *i.e.*, one scan for each of the 4 treatments mentioned above for the AIMs scoring (on Day 43, 45, 50 & 52 post-6-OHDA lesion). Rats were fasted for 4 h before each scan. Fifteen minutes after *i.p.* injection of L-DOPA,NLX-112, L-DOPA + NLX-112 or saline, [^18^F]FDG (36.4 ± 3.1 kBq/g) was injected under awake conditions. Thirty min later, rats were anesthetized with constant insufflation of isoflurane (4% for induction then 2% during acquisition, 1 L/min) and placed in the PET-CT imaging scanner (INVEON®, Siemens). PET scans were acquired in list mode during 30 min, completed by a computed tomography (CT) scan. Static images of the 30 min acquisitions were reconstructed with attenuation and scatter corrections.

#### 2.6.2 PET data analyses

Data acquisition and processing was carried out using INVEON Research Workplace (IRW®, Siemens) software and the statistical parametric mapping software SPM12®. Images were analyzed in two steps, *i.e.*, a voxel-based analysis and a regions of interest (ROIs) analysis.

### 2.7 Voxel-based analyses (VBA)

Individual PET images were realigned and spatially normalized based on our own previously built CT template, co-registered on an anatomical MRI template. Each PET volume with voxel size 0.1 × 0.1x 0.2 mm^3^ was then spatially normalized and smoothed using an isotropic Gaussian filter [1 × 1 × 1 mm^3^]. Local voxel activities were normalized to the mean uptake of the whole brain taken individually as a volume of interest (VOI), in order to minimize potential confounding factors such as changes in peripheral glucose metabolism. Indeed, the dyskinetic behavior can indirectly decrease the global brain uptake by increasing the uptake in the muscles and confirmed by SUV measurements (see supplementary Figs. 2 and 3). For each group (HPK-non-LID and HPK-LID separately), a voxel-to-voxel statistical analysis was performed, using a variance analysis in a generalized linear model, to compare mean uptakes between the different conditions. A significant threshold was set up at p < 0.001 uncorrected, at the voxel level. A second analysis was performed in order to compare the metabolic profiles of HPK-LID and HPK-non-LID rats under saline condition by using an unpaired Student’s *t*-test, in order to assess the impact of dyskinesia (chronic administration of L-DOPA) on the absorption ratios of [^18^F]FDG.

### 2.8 Regions of interest (ROIs) analyses

Brain ROIs were already defined in the Lancelot rat brain atlas on the corresponding MRI template (Lancelot et al., 2014). Mean uptake ratios were extracted in the different ROIs, and a two-way mixed ANOVA for repeated measures (factors: treatment and ROI) with Tukey’s post-hoc tests (corrected for multiple comparisons) were performed using GraphPad Prism 8.0 software to compare the [^18^F]FDG uptake ratios between the different conditions.

### 2.9 Functional MRI imaging procedures and data analysis

#### 2.9.1 fMRI protocol and image acquisition

Out of the 31 rats that were retained following the apomorphine challenge (see above), the remaining 16 (8 HPK-non-LID and 8 HPK-LID) rats were subjected to an fMRI study, 3 weeks after the apomorphine-induced rotational test. Anesthesia induction was performed with 4% isoflurane (1 L/min), then lowered to 2% isoflurane during the catheterization procedure and fMRI acquisition. Saline, NLX-112, L-DOPA or L-DOPA + NLX-112 (same doses as for the PET/scan experiments) were injected *i.p.* over one minute during the fMRI scan. The fMRI protocol was carried out on a 7-Tesla Bruker Biospec MR system (Bruker BiospinGbmH, Germany). A 2D anatomical T2-RARE (Rapid Acquisition with Relaxation Enhancement) image was obtained with 14 contiguous slices of 1.5-mm thickness covering the whole rat brain. To measure BOLD variations, a T2* (Echo Planar Imaging) sequence was used and fourteen slices acquired with the identical geometry of the anatomical T2-RARE scan. Each fMRI session lasted 1 h, corresponding to 1200 repetitions. Drugs were injected 15 min after the beginning of the session, resulting in 45 min of post-injection period (900 repetitions).

#### 2.9.2 fMRI data analyses

Data were preprocessed using the SPM12® software. Images were realigned and spatial normalization was performed using the standardized MRI template (Lancelot et al., 2014). For each acquisition, the time courses of the BOLD signal were extracted from different regions of interest (ROIs) on the 900 post-injection images. These regions were automatically delineated using the Lancelot rat brain atlas (Lancelot et al., 2014) defined in the MRI template space (Lancelot et al., 2014). Temporal correlations were evaluated by the Pearson correlation coefficient between the BOLD time-courses of each pair of ROIs over the 45 min after injection. The correlation matrix displaying the correlation coefficients for every pair of regions was generated using the Graphpad Prism 8.0 software. Matrices were averaged to obtain mean correlation matrices for each group. For statistical analysis, correlation coefficients of each acquisition were converted into Z-scores by Fisher transformation. For HPK-non-LID and HPK-LID rats, each drug condition was compared to the saline condition using a two-way ANOVA followed by Dunnett's multiple comparisons post-hoc tests using the Graphpad Prism 8.0 software.

## 3 Results

**Fig. 1:**
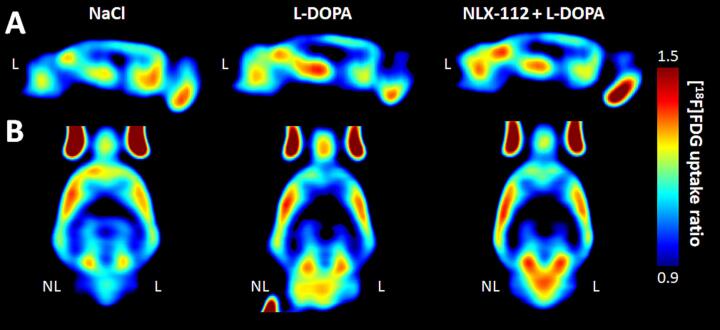
Averaged images of [^18^F]FDG PET uptake ratio in HPK-LID rats (n = 8) obtained with raw data. Comparison of three of the different treatment conditions (NaCl, L-DOPA and NLX-112 + L-DOPA). A. Sagittal section, B. Cross-Section, NL = non-lesioned side; L = lesioned side.

[^18^F]FDG uptake ratios were compared between the different groups and drug treatments. They are illustrated in Fig. 1 that shows a comparison of the averaged images of [^18^F]FDG uptake ratio after administration of saline, L-DOPA or L-DOPA + NLX-112 (n = 8 per condition).

### 3.1 Effects of acute challenge with L-DOPA and NLX-112 on AIMs in HPK-LID and HPK-non-LID rats

Upon administration of an acute *i.p.* challenge with L-DOPA/benserazide, HPK-LID rats develop high AIMs scores (average ± SEM: 2.71 ± 0.18), *i.e.* presented orolingual and moderate to strong limb & axial dyskinesia. By contrast, HPK-non-LID rats exhibit low AIMs score (1.33 ± 0.21), *i.e.*, presented mainly a normal behavior or some orolingual dyskinesia. Co-administration of NLX-112 (at 0.16 mg/kg *i.p.,* a dose that produces no dyskinesia by itself) fully reversed the AIMs elicited by L-DOPA in both cohorts of rats.

### 3.2 Effects of saline on imaging profiles of HPK-non-LID and HPK-LID

#### 3.2.1 PET imaging

**Fig. 2:**
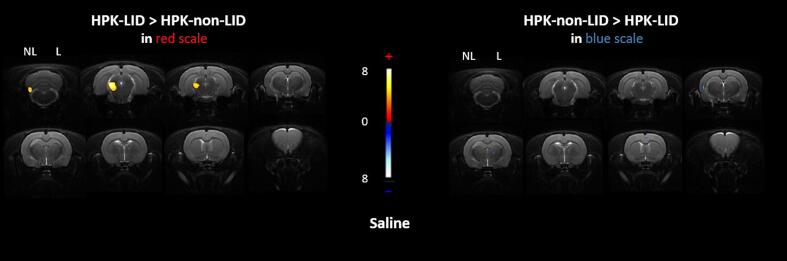
Comparison of metabolic profiles using [^18^F]FDG PET scans between HPK-non-LID and HPK-LID rats following an acute *i.p*. saline injection. Voxel-to-voxel statistical comparisons of [^18^F]FDG uptake ratio between saline injections in HPK-non-LID (n = 7) and HPK-LID (n = 8). T scores are represented in color scales (significant increases from red to light yellow, significant decreases from dark to light blue; p < 0.01). Left panel: significant increase of glucose metabolism in HPK-LID rats compared to HPK-non-LID rats; Right panel: decrease of glucose metabolism in HPK-LID rats compared to HPK-non-LID rats. HPK: hemi-parkinsonian, LID: L-DOPA-induced dyskinesia, NL = non-lesioned side, L = lesioned side. Coronal sections are from + 4 to −13 mm with respect to Bregma.

Images of voxel-based analyses of [^18^F]FDG uptake ratios in HPK-non-LID and HPK-LID rats following an acute saline injection are shown in Fig. 2. The metabolic profiles of HPK-non-LID and HPK-LID rats are identical in terms of [^18^F]FDG uptake ratios, except for hypermetabolism in the mesencephalic locomotor region on the contralateral lesion side of HPK-LID rats.

### 3.3 Effects of L-DOPA on imaging profiles of HPK-non-LID and HPK-LID

**Fig. 3:**
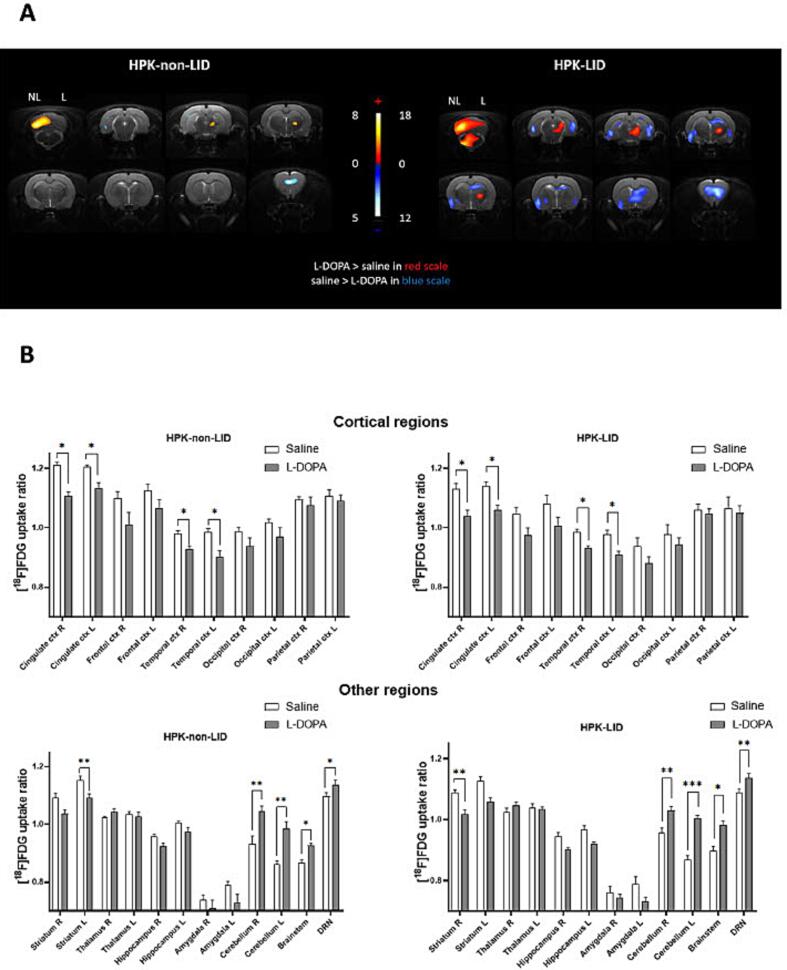
Comparison of metabolic profiles using [^18^F]FDG PET scans between HPK-non-LID and HPK-LID rats after an acute challenge administration of saline or L-DOPA (6 mg/kg, *i.p.*). A- Voxel-to-voxel statistical comparisons of [^18^F]FDG uptake ratio between L-DOPA injections and saline in HPK-non-LID rats (left panel; n = 7) and in HPK-LID rats (right panel; n = 8). T scores are represented in color scales (significant increases from red to light yellow, significant decreases from dark to light blue; p < 0.01). HPK: hemi-parkinsonian, LID: L-DOPA-induced dyskinesia, NL = non-lesioned side, L = lesioned side. Coronal sections are from + 4 to −13 mm with respect to Bregma. B- [^18^F]FDG uptake ratio in several ROIs after saline or L-DOPA injection in HPK-LID (n = 8) and HPK-non-LID rats (n = 7). Bars are the mean + SEM; * p < 0.05, ** p < 0.01, *** p < 0.001, Tukey’s multiple comparisons test, following two-way ANOVAs; for top panel (HPK-non-LID): region factor: F (2.564, 15.38) = 58.05, p < 0.0001; treatment factor: F (2.162, 12.97) = 5.043, p = 0.0222; interaction factor: F (2.930, 16.56) = 3.135, p = 0.0545; for bottom panel (HPK-LID): region factor: F (3.407, 23.85) = 85.99, p < 0.0001; treatment factor: F (2.309, 16.16) = 7.055, p = 0.0049; interaction factor: F (4.054, 28.38) = 4.092, p = 0.0094. L = left (non-lesioned side), R = right (lesioned side).

Fig. 3A represents the voxel-to-voxel statistical comparisons of [^18^F]FDG uptake ratios between L-DOPA/benserazide and saline injections in HPK-non-LID and HPK-LID rats. The metabolic pattern of activation in HPK-LID rats is more pronounced than that of HPK-non-LID rats after L-DOPA acute administration. Indeed, significant activations were detected in the cerebellum, brainstem, raphe area and thalamus, on the unilateral side of the lesion in HPK-LID rats. Significant clusters of hypometabolism were found in the hippocampus, the ipsilateral striatum and in the cortex, especially the prefrontal cortex.

Results from the ROIs analysis in HPK-non-LID and HPK-LID rats (Fig. 3B) also showed a significant increase of metabolism in the brainstem in HPK-non-LID (+7%) and HPK-LID rats (+9.5%) as well as in the right (+12.1%) and left (+14.4%) cerebellum in HPK-non-LID rats and in HPK-LID rats (+7.6% and + 15.8%). A significant increase was found in the raphe nuclei in HPK-non-LID rats (+3.6%), more pronounced in HPK-LID rats (+4.4%).

Concerning [^18^F]FDG uptake ratio in HPK-LID rats, the results are also representative of the images of voxel-based analysis. A decrease was detected in the right (-6.6%) and in some cortical regions compared to saline injection in HPK-LID rats. The results were similar concerning HPK-non-LID rats. A decrease was observed in the left striatum (-5.3%) and in some cortical regions: −8.6% and −6.0% for right and left cingulate cortex and −5.4% and −8.7% for right and left temporal cortex.

#### 3.3.1 fMRI imaging functional connectivity

**Fig. 4:**
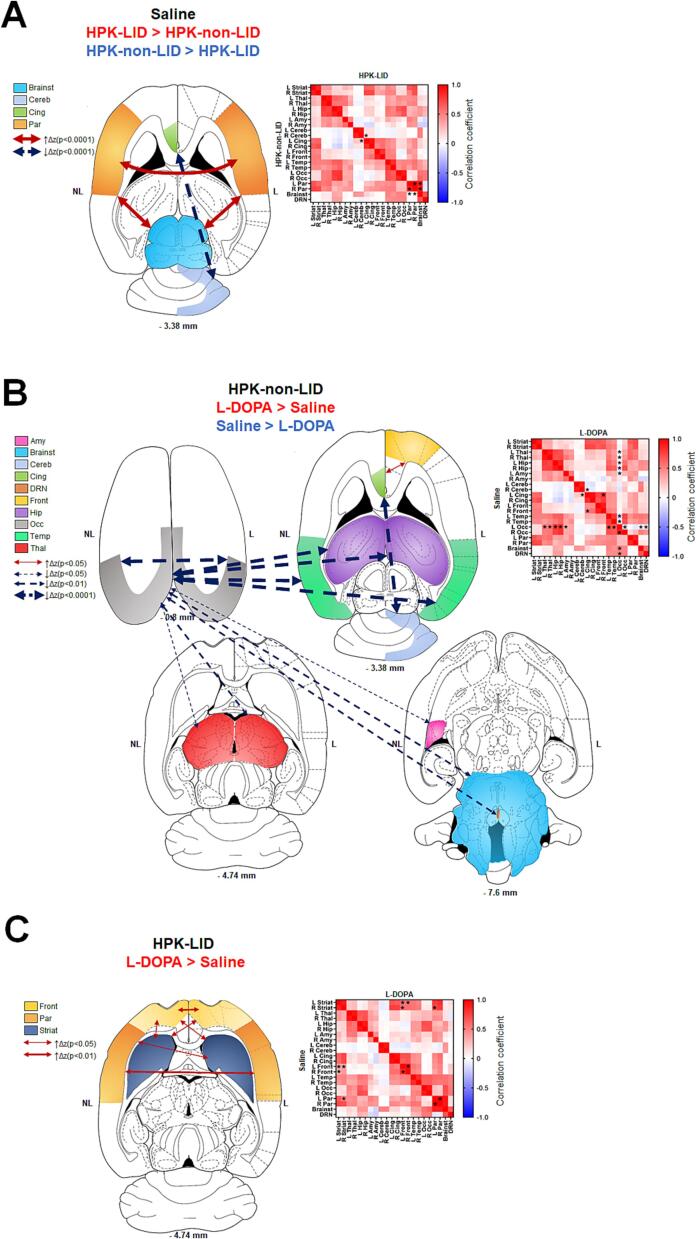
Functional connectivity analysis using fMRI between HPK-non-LID (n = 7) and HPK-LID rats (n = 8) following a saline *i.p.* catheter infusion. A- Analysis following injection of saline between HPK-LID rats and HPK-non-LID rats. B- Analysis between L-DOPA and saline injections in HPK-non-LID rats. C- Analysis between L-DOPA and saline injections in HPK-LID rats. Schematic representations (on the left) of the significant changes in functional connectivity (p < 0.001) as measured by comparing the correlation coefficient after Fisher transformation of r values into z values. The dashed arrows represent the significant decreases of functional connectivity between two regions and the solid arrows represent the significant increases of functional connectivity between two regions, as compared to the saline. The correlation matrices (on the right) are expressed as mean values of Pearson correlation coefficient. Positive values of correlation coefficient are shown in red and negative values of correlation coefficient are shown in blue. For statistical comparisons, correlation coefficients were transformed into Z-scores using Fisher transformation before two-way ANOVA, followed by Sidak (in A) or Dunnett (in B and C) multiple comparisons post-hoc test (*p < 0.05, ** p < 0.01, **** p < 0.0001, HPK-LID vs HPK-non-LID (in A), or L-DOPA versus saline in HPK-non-LID (in B) or in HPK-LID (in C)).

The development of LID produced changes in functional connectivity following acute administration of saline (Fig. 4A) and acute administration of L-DOPA (Fig. 4B and Fig. 4C). There was a statistically significant effect of the pairs of regions and the interaction between the pairs of regions and the groups on the Z-score. Post-hoc tests revealed increases of connectivity in HPK-LID rats compared to HPK-non-LID rats between the brainstem and the parietal cortex and between the parietal cortex of the right and left hemispheres. In contrast, a decrease between the left cingulate cortex (non-lesioned side) and the right cerebellum (lesioned side) was observed.

Functional connectivity was also compared between the L-DOPA and the saline injections in each cohort of rats using two-way ANOVAs. In HPK-non-LID rats, there was a statistically significant effect of the pairs of regions, the drug conditions and their interaction on the Z-scores. Post-hoc tests revealed that following acute administration of L-DOPA in HPK-non-LID rats (Fig. 4B), a strong and specific loss of connectivity was found between the left occipital cortex (non-lesioned side) and many other brain structures (thalamus, hippocampus, temporal cortex, left amygdala, brainstem, and raphe). When compared with saline treatment, there was also a disconnection between the lesioned and non-lesioned side in the occipital cortex. Concurrently, other significant decreases of connectivity were found between the left cingulate cortex and the right cerebellum. A connectivity increase between the right frontal cortex and left cingulate cortex was also observed.

In HPK-LID rats, there was a statistically significant effect of the pairs of regions, the drug conditions but not their interaction on the Z-scores. The post-hoc tests showed that when comparing L-DOPA and saline (Fig. 4C), the alteration of the connectivity of the left occipital cortex was no longer observed in that group. On the other hand, increases of connectivity were found between both hemispheres of the frontal cortex and the left striatum and between the right striatum and the left frontal cortex, as well as between the left parietal cortex and the right striatum (lesioned side). A significant increase of connectivity between the right and left sides of the parietal and the frontal cortices was also observed.

### 3.4 Effects of NLX-112 on L-DOPA-induced activity in LID rats

#### 3.4.1 PET imaging

**Fig. 5:**
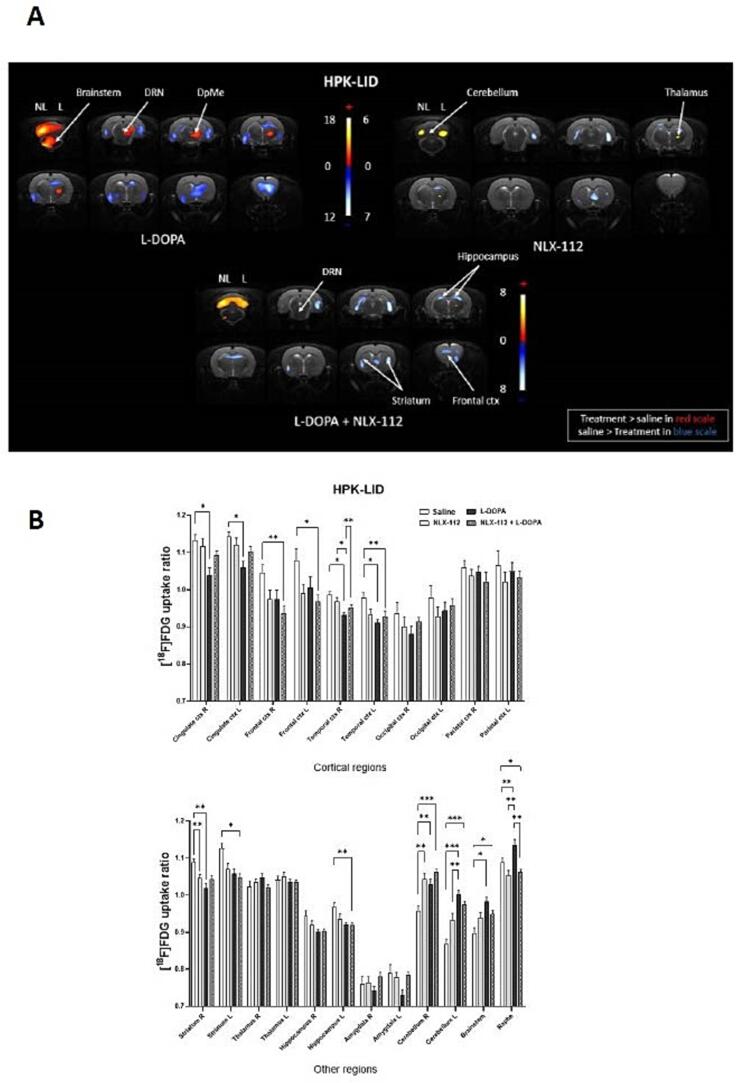
Effects of administration of NLX-112 at 0.16 mg/kg on glucose metabolism using [^18^F]FDG PET scans in HPK-LID rats. A - Voxel-to-voxel statistical comparisons of [^18^F]FDG uptake ratio in HPK-LID rats between L-DOPA alone and saline injections in HPK-LID rats (on the left; n = 8); NLX-112 alone at an antidyskinetic dose (0.16 mg/kg) and saline injections (on the right; n = 8); co-administration NLX-112 + L-DOPA and saline (below; n = 8). T scores are represented in color scales (significant increases from red to light yellow, significant decreases from dark to light blue; p < 0.01.HPK: hemi-parkinsonian, LID: L-DOPA-induced dyskinesia, NL = non-lesioned side, L = lesioned side. Coronal sections are from + 4 to −13 mm with respect to Bregma. Note that L-DOPA alone data are the same as those already presented in Fig. 3. B - [^18^F]FDG uptake ratio in several ROIs after L-DOPA, saline injection, NLX-112 alone or co-administration of NLX-112 + L-DOPA in HPK-LID rats (n = 8). Bars are the mean + SEM; * p < 0.05, ** p < 0.01, *** p < 0.001, Tukey’s multiple comparisons test, following two-way ANOVAs. L = left (non-lesioned side), R = right (lesioned side).

Images of voxel-based analysis of the effects of NLX-112 on glucose metabolism in HPK-LID animals are shown in Fig. 5A. By itself, NLX-112 produced an increase in glucose metabolism in cortical areas and a decrease in motor regions (thalamus, cerebellum, brainstem) and raphe nuclei. Co-administration of L-DOPA and NLX-112 partially erased the cerebral effects of L-DOPA in some, but not all, brain regions (Fig. 5A). In particular, voxel-to-voxel comparisons suggested that NLX-112 reverses the L-DOPA-induced hypometabolism observed in cortical regions and reduces the L-DOPA-induced hypermetabolism seen in brainstem and cerebellum (Fig. 5A). These effects were echoed in the uptake ratios (Fig. 5B) which also found a robust normalization by NLX-112 of [^18^F]FDG uptake in raphe nuclei, a main target region underlying NLX-112′s therapeutic activity.

#### 3.4.2 Functional connectivity

**Fig. 6:**
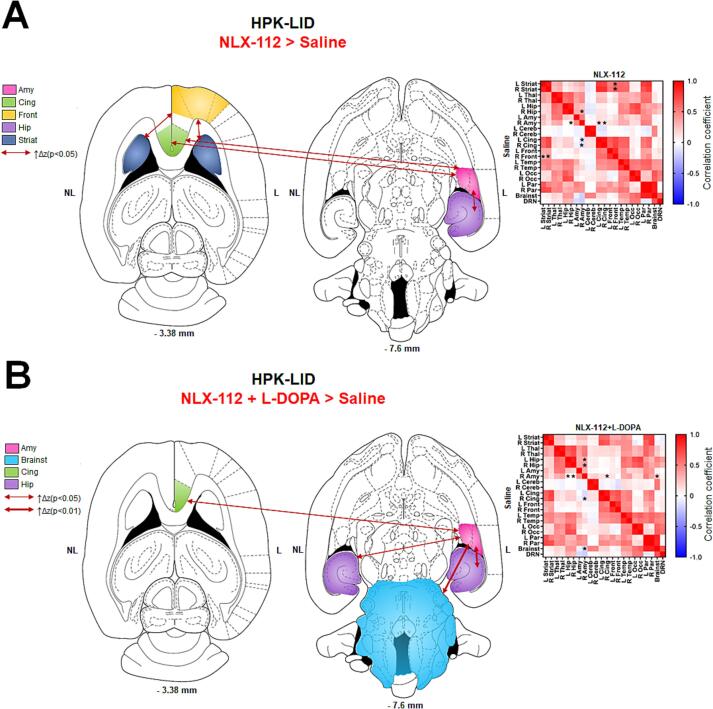
Effects of administration of NLX-112 at 0.16 mg/kg on functional connectivity using fMRI in HPK-LID rats. A - Functional connectivity analysis between NLX-112 alone and saline *i.p.* catheter infusions in HPK-LID rats (n = 8) and B - between co-administration of L-DOPA + NLX-112 injections and saline in HPK-LID rats (n = 8). Schematic representations (on the left) of the significant changes in functional connectivity (p < 0.001) as measured by comparing the correlation coefficient after Fisher transformation of r values into z values. The dashed arrows represent the significant decreases of functional connectivity between two regions and the solid arrows represent the significant increases of functional connectivity between two regions, as compared to the saline. The correlation matrices (on the right) are expressed as mean values of Pearson correlation coefficient. Positive values of correlation coefficient are shown in red and negative values of correlation coefficient are shown in blue. For statistical comparisons, correlation coefficients were transformed into Z-scores using Fisher transformation before two-way ANOVA followed by Dunnett’s multiple comparisons test (*p < 0.05, ** p < 0.01, **** p < 0.0001).

NLX-112 at a therapeutic dose (0.16 mg/kg) produced numerous changes in functional connectivity in HPK-LID rats (Fig. 6). Post-hoc tests following two-way ANOVAs in HPK-LID rats showed that NLX-112 alone (Fig. 6A) produced connectivity increases between the right amygdala and the right hippocampus), between both sides of the cingulate cortex and between the left and right striatum and the right frontal cortex. The co-administration of NLX-112 with L-DOPA (Fig. 6B) prevented the functional hyper-connectivity at the corticostriatal level that was observed with L-DOPA alone. Significant increases of connectivity were found between the right amygdala and brainstem, right cingulate cortex and left and right hippocampus.

### 3.5 Effects of NLX-112 on L-DOPA-induced activity in non-LID rats

The metabolic and functional connectivity changes occurring after NLX-112 injection in non-LID animals were also evaluated (Supplementary Fig. 4). NLX-112 alone produced few effects on [^18^F]FDG uptake, but prevented the frontal decrease and attenuated the cerebellar increase induced by L-DOPA. NLX-112 alone also produced few effects on the functional connectivity in non-LID rats, but co-administration with L-DOPA was followed by numerous decreases of connectivity between various subcortical regions and the occipital cortex, as observed with L-DOPA alone, accompanied by a bilateral increase of connectivity between the frontal cortex, the cingulate cortex and the hippocampus.

## 4 Discussion

### 4.1 Validity of the rat HPK-LID model

**Supplementary figure 1:**
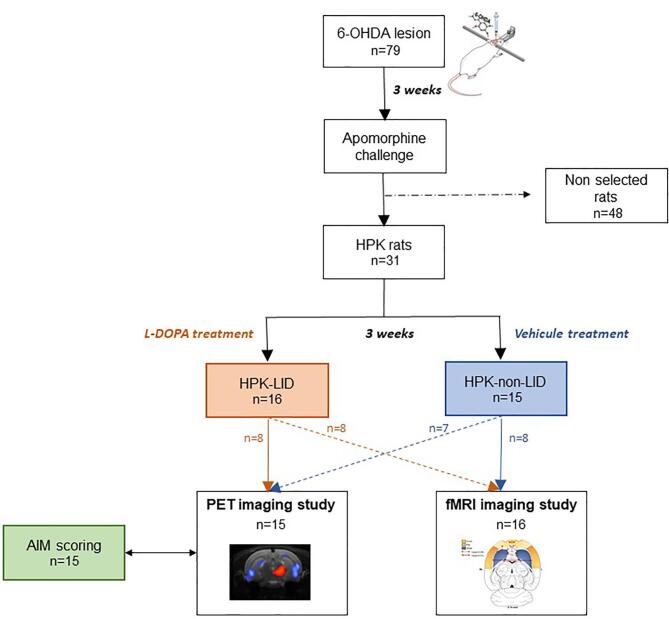
Study design

**Supplementary figure 2:**
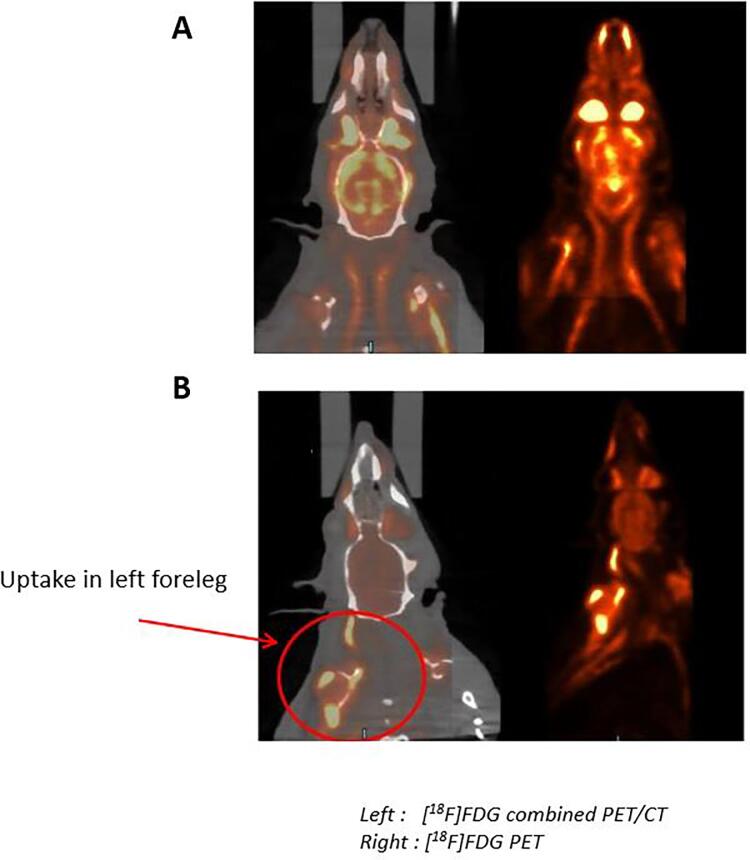
Comparison of [^18^F]FDG uptake in the same HPK-LID rat after saline (A) or L-DOPA (B) injection (raw image). These images show an increased radiotracer uptake in the left foreleg (opposite side to the lesioned side) after L-DOPA injection.Left: [^18^F]FDG combined PET/CT, Right : [^18^F]FDG PET

**Supplementary figure 3:**
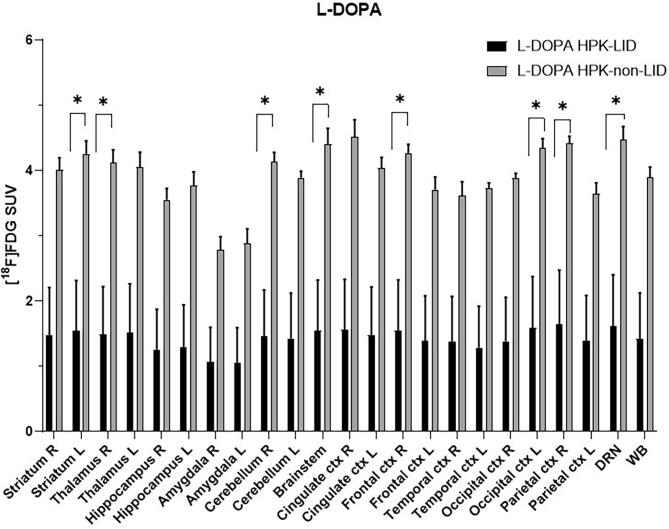
Comparison of SUV values using [^18^F]FDG PET scans between HPK-non-LID and HPK-LID rats after an acute challenge administration of saline or L-DOPA (6 mg/kg, i.p.). [^18^F]FDG PET mean SUVs in several ROIs after L-DOPA injection in HPK-LID (n=8) and HPK-non-LID rats (n=7). Bars are the mean + SEM; * p < 0.05. Sidak’s multiple comparisons test, following two-way ANOVAs; region factor: F (22, 276) = 0.1303, p>0.9999; group factor: F (22, 276) = 0.5027, p=0.9713; interaction factor: F (1, 276) = 201.2, p<0.0001; L = left (non-lesioned side), R= right (lesioned side). DRN = Dorsal Raphe Nuclei, WB = whole-brain

**Supplementary figure 4:**
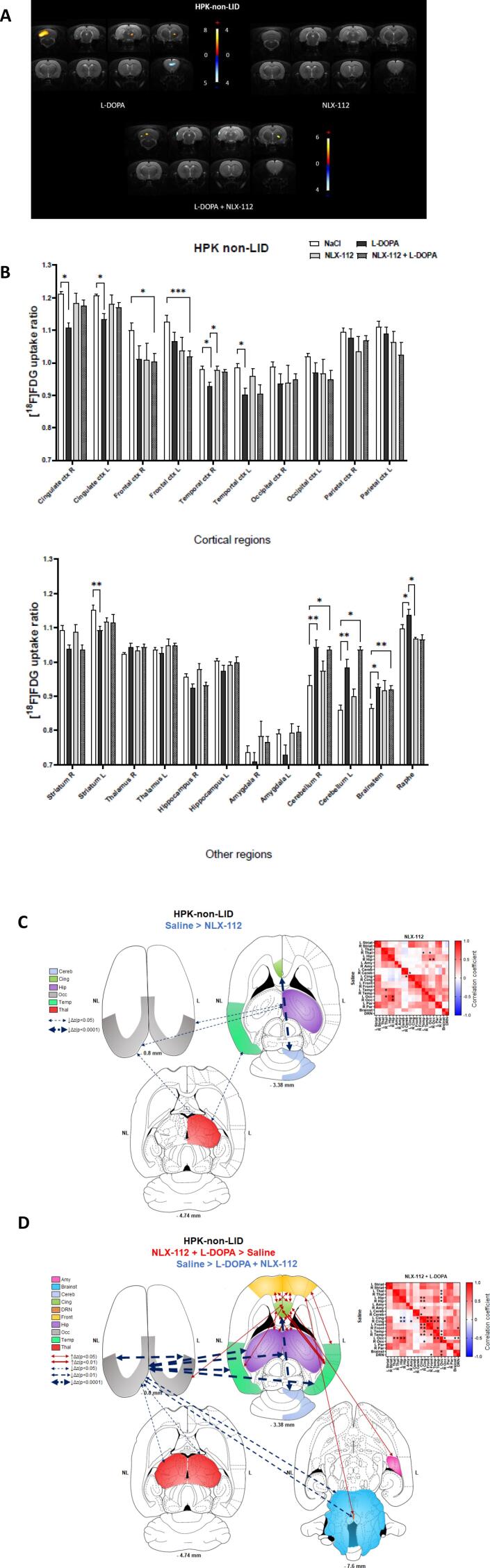
Effects of administration of NLX-112 at 0.16 mg/kg on glucose metabolism using [^18^F]FDG PET scans in HPK-non-LID rats following acute i.p. administration of saline or L-DOPA. A - Voxel-to-voxel statistical comparisons of [^18^F]FDG uptake ratio in HPK-non-LID rats between L-DOPA alone and saline injections in HPK-LID rats (on the left; n=7) ; between NLX-112 alone at 0.16 mg/kg and saline injections (on the right; n=7); between co-administration NLX-112 + L-DOPA and saline  (bottom; n=7). T scores in color scales (significant increases of glucose metabolism in red and significant decreases in blue; p<0.01, Student’s t test). HPK: hemi-parkinsonian, LID: L-DOPA-induced dyskinesia, NL = non-lesioned side, L = lesioned side. Coronal sections are from +4 to -13 mm with respect to Bregma.  B - [^18^F]FDG uptake ratio in several ROIs after L-DOPA, saline injection, NLX-112 alone or co-administration of NLX-112 + L-DOPA in HPK non-LID rats (n=7). Bars are the mean + SEM; * p < 0.05, ** p < 0.01, *** p < 0.001, Tukey’s multiple comparisons test, following two-way ANOVAs. Region factor: F (2.662, 15.97) = 54.49, p<0.0001; treatment factor: F (2.002, 12.01) = 5.838, p=0.0169; interaction factor: F (2.977, 16.83) = 2.939, p=0.0636. L = left (non-lesioned side), R= right (lesioned side), Ctx = cortex  C- Functional connectivity analysis between NLX-112 alone and saline in HPK-non-LID rats (n=8) and D- between co-administration of L-DOPA + NLX-112 and saline in HPK-non-LID rats (n=8). Schematic representations (on the left) of the significant changes in functional connectivity (p < 0.001) as measured by comparing the correlation coefficient after Fisher transformation of r values into z values. The dashed arrows represent the significant decreases of functional connectivity between two regions and the solid arrows represent the significant increases of functional connectivity between two regions, as compared to the saline. The correlation matrices (on the right) are expressed as mean values of Pearson correlation coefficient. Positive values of correlation coefficient are shown in red and negative values of correlation coefficient are shown in blue. For statistical comparisons, correlation coefficients were transformed into Z-scores using Fisher transformation before two-way ANOVA followed by Dunnett’s multiple comparisons test (*p < 0.05, ** p < 0.01, **** p < 0.0001).

The present neuroimaging study describes, for the first-time, specific brain metabolic patterns of LID produced by an acute injection of L-DOPA and/or the 5-HT_1A_R biased agonist, NLX-112, in vigil HPK rats with unilateral 6-OHDA lesions of the substantia nigra. This animal model of PD has the benefit of being robust, reproducible and widely described, with a lesion success rate of at least 50% (Hudson et al., 1993) and a good tolerance to the unilateral lesion (Schwarting and Huston, 1996). The HPK-LID rat model presents similar characteristics to “peak dose dyskinesia” (good face validity) and responds to antidyskinetic treatments (good predictive validity). About 80% of rodents with a DAergic lesion greater than 90% rapidly develop dyskinesia (Cenci and Lundblad, 2007). Indeed, with this methodology we found a massive unilateral decrease of the dopamine transporter binding in an additional cohort of rats (Supplementary Fig. 1).

### 4.2 [^18^F]FDG PET imaging in HPK-LID rats

Changes in glucose uptake ratios revealed by [^18^F]FDG PET imaging reflect cerebral activity dynamics (Wehrl et al., 2013). Thus, in the basal state (*i.e.*, after saline injection), HPK-non-LID and HPK-LID rats had generally similar metabolic profiles (Fig. 2), but clear differences in brain activity were observed after acute L-DOPA challenge (Fig. 3A), which confirms the “peak-dose” aspect of the dyskinesia obtained in HPK-LID rats (Cenci and Crossman, 2018, de la Fuente-Fernández et al., 2004). The metabolic pattern elicited by L-DOPA in HPK-LID rats was characterized by decreased glucose metabolism in the cingulate and temporal cortex and the ipsilateral striatum. In contrast, glucose hypermetabolism was found in the dorsal raphe nuclei, cerebellum, brainstem, regions that are involved in motor control and influence basal ganglia activity (Bezard et al., 2001).

Interestingly, the regions mentioned above are similar to those affected in [^18^F]FDG PET studies of PD patients and are referred to as the “PD-related pattern” (PDRP), with a hypermetabolism in the cerebellum, pons, thalamus and lentiform nucleus and a hypometabolism in premotor and parietal cortex (Eidelberg et al., 1994, Ma et al., 2007). In contrast, parkinsonian tremor seems to have its own pattern in humans, with an increase in metabolic activity in the cerebellum, pons, primary motor cortex, caudate nucleus and putamen (Mure et al., 2011).

In our HPK-LID rat model, we only found hypoactivity in the prefrontal cortex and the ipsilateral striatum. The striatal hypoactivity may be due to the effect of endogenous DA on inhibitory D_2_ receptors, since it possesses higher affinity for them than for activatory D_1_ receptors (Marcellino et al., 2012). Furthermore, the activating effects of L-DOPA in the raphe nuclei point to the involvement of the serotonergic system in LID. This is consistent with the role of serotonergic neurons in converting L-DOPA to DA and releasing it as a “false neurotransmitter” (Carta and Bezard, 2011, Cenci, 2014).

### 4.3 Functional connectivity in HPK-LID rats

The functional connectivity experiments provide further evidence of the alteration of basal ganglia circuitry in PD (Perlbarg et al., 2018) and in the development of LID (Wichmann and Delong, 2006) and are consistent with functional connectivity studies carried out in humans. In particular, aberrant cortico-striatal changes in connectivity are found between cortical areas and the putamen (Herz et al., 2016, Tessitore et al., 2019). In addition, a correlation is observed between the connectivity changes in the prefrontal cortex and the occurrence of AIMs (Cerasa et al., 2016, Cerasa et al., 2013). A major involvement of the cerebellum is also found in human with an increase in connectivity between it and other regions (Mueller et al., 2019, Tessitore et al., 2019).

Interestingly, a marked decrease of connectivity between other cerebral structures and the left occipital cortex was found in HPK-non-LID rats with acute L-DOPA administration. This disconnection was not found in dyskinetic rats and may be correlated with the known occipital hypoperfusion in treated parkinsonian patients (Abe et al., 2003). Another study reported an alteration of fMRI resting-state measurements in the occipital cortex which was correlated with decreased DA-uptake by the DA transporter in the caudate, showing the link between this region and the striatal dopaminergic system (Oh et al., 2020).

### 4.4 NLX-112 as a promising anti-dyskinetic drug for LID in Parkinson’s disease?

Beyond the imaging phenotyping of the HPK-LID model, the second objective of this study was to investigate the central effects of NLX-112, a selective 5-HT_1A_R biased agonist. Administration of NLX-112 completely prevented L-DOPA-induced AIMs in HPK-LID rats, confirming the antidyskinetic activity of the molecule (Depoortere et al., 2020, Fisher et al., 2020, Iderberg et al., 2015, McCreary et al., 2016). The behavioral effects of NLX-112 were accompanied by a partial reversal of the metabolic changes induced by L-DOPA: thus, NLX-112 restored cortical metabolism and completely reversed the hypermetabolism caused by L-DOPA in the raphe nuclei, reducing it in the mesencephalic motor region, brainstem, and cerebellum. The metabolic profile observed upon combined NLX-112 and L-DOPA administration did not, however, result in a complete return to a basal state and the remaining changes may be due to the intrinsic 5-HT_1A_ agonist activity of NLX-112, as characterized previously in non-lesioned rats (Vidal et al., 2020).

Furthermore, administration of NLX-112 corrected the functional connectivity profile in HPK-LID rats treated with L-DOPA. Taking these observations together with previous behavioral data in 6-OHDA-lesioned rats (Iderberg et al., 2015), it would appear that the main mechanism for the antidyskinetic effects of NLX-112 is a preferential stimulation of somatodendritic 5-HT_1A_ autoreceptors in the raphe nuclei. This activation decreases the activity of the ascending serotonergic system and inhibits the excessive release of DA by 5-HT terminals (Iderberg et al., 2015, Lladó-Pelfort et al., 2012, Muñoz et al., 2008, Ohno et al., 2013).

## 5 Conclusion

This study defined for the first time the brain activation and inhibition patterns in functional [^18^F]FDG PET imaging and the functional connectivity profiles of HPK rats presenting dyskinesia after chronic administration of L-DOPA. These data enrich the limited knowledge available concerning the functional imaging of LID. Moreover, the present observations further reinforce the involvement of the serotonin system in LID (Huot et al., 2017) and support targeting 5-HT_1A_R as a therapeutic strategy for LID treatment (Huot, 2018, Muñoz et al., 2008, Ohno et al., 2013). Finally, these preclinical results obtained by translational imaging approaches are directly transferable to human studies and inform ongoing clinical development of NLX-112 in Parkinson's disease patients suffering from LID.

## 6 Authors’ Roles

Sarah Chaib carried out the experiments with PET imaging, performed data analysis, participated in the study design, and wrote the manuscript.

Benjamin Vidal carried out the experiments with fMRI imaging and autoradiography, performed data analysis, participated in the study design and reviewed the manuscript.

Caroline Bouillot carried out the experiments with PET imaging.

Ronan Depoortere participated in the behavioral analysis of the study and reviewed the manuscript.

Adrian Newman-Tancredi provided NLX-112 and reviewed the manuscript.

Luc Zimmer initiated the study, participated in its design and reviewed the manuscript.

Elise Levigoureux coordinated the study, carried out the experiments, participated in its design and reviewed the manuscript.

All authors have read and approved the final manuscript.

## Funding sources for study

This research received no specific grant from any funding agency in the public, commercial, or not-for-profit sectors.

## Financial disclosures

Dr. Adrian Newman-Tancredi and Dr Ronan Depoortere are employees and stockholders of Neurolixis. The other authors report no conflict of interest and have nothing to disclose.

## Declaration of Competing Interest

The authors declare that they have no known competing financial interests or personal relationships that could have appeared to influence the work reported in this paper.

## Figures and Tables

**Supplementary figure 5:**
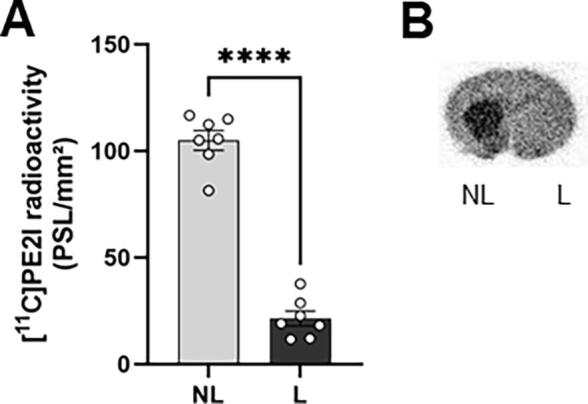
Validation of the hemiparkinsonian rat model (HPK) in an additional cohort of n=7 rats (selected using the same criterion defined for the rats used in the imaging experiments, with the apomorphine challenge) using autoradiography with [^11^C]PE2I, a PET radiotracer of dopamine transporters (DAT). All animals show a strong striatal depletion of DAT in the lesioned side compared to the non-lesioned side (mean depletion of -83.52%, p<0.0001, paired t-test). A. Results expressed in optical densities (PSL/mm^2^) and B. Difference between the lesioned side (L) and the non-lesioned side (NL) of binding on a rat brain section.

## Data Availability

Data will be made available on request.

## References

[b0005] Abe Y., Kachi T., Kato T., Arahata Y., Yamada T., Washimi Y., Iwai K., Ito K., Yanagisawa N., Sobue G. (2003). Occipital hypoperfusion in Parkinson’s disease without dementia: correlation to impaired cortical visual processing. J. Neurol. Neurosurg. Psychiatry.

[b0010] Bartoszyk G.D., van Amsterdam C., Greiner H.E., Rautenberg W., Russ H., Seyfried C.A. (2004). Sarizotan, a serotonin 5-HT 1A receptor agonist and dopamine receptor ligand. 1. Neurochemical profile. J. Neural Transm..

[b0015] Bastide M.F., Meissner W.G., Picconi B., Fasano S., Fernagut P.-O., Feyder M., Francardo V., Alcacer C., Ding Y., Brambilla R., Fisone G., Jon Stoessl A., Bourdenx M., Engeln M., Navailles S., De Deurwaerdère P., Ko W.K.D., Simola N., Morelli M., Groc L., Rodriguez M.-C., Gurevich E.V., Quik M., Morari M., Mellone M., Gardoni F., Tronci E., Guehl D., Tison F., Crossman A.R., Kang U.J., Steece-Collier K., Fox S., Carta M., Angela Cenci M., Bézard E. (2015). Pathophysiology of L-dopa-induced motor and non-motor complications in Parkinson’s disease. Prog. Neurobiol..

[b0020] Bezard E., Brotchie J.M., Gross C.E. (2001). Pathophysiology of levodopa-induced dyskinesia: potential for new therapies. Nat. Rev. Neurosci..

[b0025] Bezard E., Tronci E., Pioli E.Y., Li Q., Porras G., Björklund A., Carta M. (2013). Study of the antidyskinetic effect of eltoprazine in animal models of levodopa-induced dyskinesia. Mov. Disord. Off. J. Mov. Disord. Soc..

[b0030] Bishop C., Krolewski D.M., Eskow K.L., Barnum C.J., Dupre K.B., Deak T., Walker P.D. (2009). Contribution of the striatum to the effects of 5-HT1A receptor stimulation in L-DOPA-treated hemiparkinsonian rats. J. Neurosci. Res..

[b0035] Carta M., Bezard E. (2011). Contribution of pre-synaptic mechanisms to L-DOPA-induced dyskinesia. Neuroscience.

[b0040] Carta M., Carlsson T., Kirik D., Björklund A. (2007). Dopamine released from 5-HT terminals is the cause of L-DOPA-induced dyskinesia in parkinsonian rats. Brain J. Neurol..

[b0045] Cenci M.A. (2014). Presynaptic Mechanisms of l-DOPA-Induced Dyskinesia: The Findings, the Debate, and the Therapeutic Implications. Front. Neurol..

[b0050] Cenci M.A., Crossman A.R. (2018). Animal models of l-dopa-induced dyskinesia in Parkinson’s disease. Mov. Disord. Off. J. Mov. Disord. Soc..

[b0055] Cenci M.A., Lundblad M. (2007). Ratings of L-DOPA-induced dyskinesia in the unilateral 6-OHDA lesion model of Parkinson’s disease in rats and mice. Curr. Protoc. Neurosci. Chapter.

[b0060] Cerasa A., Morelli M., Augimeri A., Salsone M., Novellino F., Gioia M.C., Arabia G., Quattrone A. (2013). Prefrontal thickening in PD with levodopa-induced dyskinesias: new evidence from cortical thickness measurement. Parkinsonism Relat. Disord..

[b0065] Cerasa A., Novellino F., Quattrone A. (2016). Connectivity Changes in Parkinson’s Disease. Curr. Neurol. Neurosci. Rep..

[b0070] Colpaert F.C., Tarayre J.P., Koek W., Pauwels P.J., Bardin L., Xu X.-J., Wiesenfeld-Hallin Z., Cosi C., Carilla-Durand E., Assié M.B., Vacher B. (2002). Large-amplitude 5-HT1A receptor activation: a new mechanism of profound, central analgesia. Neuropharmacology.

[b0075] de la Fuente-Fernández R., Sossi V., Huang Z., Furtado S., Lu J.-Q., Calne D.B., Ruth T.J., Stoessl A.J. (2004). Levodopa-induced changes in synaptic dopamine levels increase with progression of Parkinson’s disease: implications for dyskinesias. Brain J. Neurol..

[b0080] de Lau L.M.L., Breteler M.M.B. (2006). Epidemiology of Parkinson’s disease. Lancet Neurol..

[b0085] Depoortere R., Johnston T.H., Fox S.H., Brotchie J.M., Newman-Tancredi A. (2020). The selective 5-HT1A receptor agonist, NLX-112, exerts anti-dyskinetic effects in MPTP-treated macaques. Parkinsonism Relat. Disord..

[b0090] Eidelberg D., Moeller J.R., Dhawan V., Spetsieris P., Takikawa S., Ishikawa T., Chaly T., Robeson W., Margouleff D., Przedborski S., Fahn S. (1994). The metabolic topography of parkinsonism. J. Cereb. Blood Flow Metab. Off. J. Int. Soc. Cereb. Blood Flow Metab..

[b0095] Eskow K.L., Gupta V., Alam S., Park J.Y., Bishop C. (2007). The partial 5-HT(1A) agonist buspirone reduces the expression and development of l-DOPA-induced dyskinesia in rats and improves l-DOPA efficacy. Pharmacol. Biochem. Behav.

[b0100] Farajdokht F., Sadigh-Eteghad S., Majdi A., Pashazadeh F., Vatandoust S.M., Ziaee M., Safari F., Karimi P., Mahmoudi J. (2020). Serotonergic system modulation holds promise for L-DOPA-induced dyskinesias in hemiparkinsonian rats: A systematic review. EXCLI J..

[b0105] Fisher R., Hikima A., Morris R., Jackson M.J., Rose S., Varney M.A., Depoortere R., Newman-Tancredi A. (2020). The selective 5-HT1A receptor agonist, NLX-112, exerts anti-dyskinetic and anti-parkinsonian-like effects in MPTP-treated marmosets. Neuropharmacology.

[b0110] Gerlach M., Bartoszyk G.D., Riederer P., Dean O., van den Buuse M. (2011). Role of dopamine D3 and serotonin 5-HT 1A receptors in L: -DOPA-induced dyskinesias and effects of sarizotan in the 6-hydroxydopamine-lesioned rat model of Parkinson’s disease. J. Neural Transm. Vienna Austria.

[b0115] Grandas F., Galiano M.L., Tabernero C. (1999). Risk factors for levodopa-induced dyskinesias in Parkinson’s disease. J. Neurol..

[b0120] Grégoire L., Samadi P., Graham J., Bédard P.J., Bartoszyk G.D., Di Paolo T. (2009). Low doses of sarizotan reduce dyskinesias and maintain antiparkinsonian efficacy of L-Dopa in parkinsonian monkeys. Parkinsonism Relat. Disord..

[b0125] Herz D.M., Haagensen B.N., Nielsen S.H., Madsen K.H., Løkkegaard A., Siebner H.R. (2016). Resting-state connectivity predicts levodopa-induced dyskinesias in Parkinson’s disease. Mov. Disord. Off. J. Mov. Disord. Soc..

[b0130] Hudson J.L., van Horne C.G., Strömberg I., Brock S., Clayton J., Masserano J., Hoffer B.J., Gerhardt G.A. (1993). Correlation of apomorphine- and amphetamine-induced turning with nigrostriatal dopamine content in unilateral 6-hydroxydopamine lesioned rats. Brain Res..

[b0135] Huot P. (2018). 5-HT1A agonists and dyskinesia in Parkinson’s disease: a pharmacological perspective. Neurodegener. Dis. Manag..

[b0140] Huot P., Sgambato-Faure V., Fox S.H., McCreary A.C. (2017). Serotonergic Approaches in Parkinson’s Disease: Translational Perspectives, an Update. ACS Chem. Nerosci..

[b0145] Iderberg H., McCreary A.C., Varney M.A., Kleven M.S., Koek W., Bardin L., Depoortère R., Cenci M.A., Newman-Tancredi A. (2015). NLX-112, a novel 5-HT1A receptor agonist for the treatment of L-DOPA-induced dyskinesia: Behavioral and neurochemical profile in rat. Exp. Neurol..

[b0150] Kuzhikandathil E.V., Bartoszyk G.D. (2006). The novel antidyskinetic drug sarizotan elicits different functional responses at human D2-like dopamine receptors. Neuropharmacology.

[b0155] Lancelot S., Roche R., Slimen A., Bouillot C., Levigoureux E., Langlois J.-B., Zimmer L., Costes N., Baron J.-C. (2014). A multi-atlas based method for automated anatomical rat brain MRI segmentation and extraction of PET activity. PLoS One.

[b0160] Lees A.J., Shaw K.M., Stern G.M. (1977). “Off period” dystonia and “on period” choreoathetosis in levodopa-treated patients with Parkinson’s disease. Lancet Lond. Engl..

[b0165] Lees A.J., Hardy J., Revesz T. (2009). Parkinson's disease. Parkinson’s disease. Lancet Lond. Engl..

[b0170] Levigoureux E., Vidal B., Fieux S., Bouillot C., Emery S., Newman-Tancredi A., Zimmer L. (2019). Serotonin 5-HT 1A Receptor Biased Agonists Induce Different Cerebral Metabolic Responses: A [18 F]-Fluorodesoxyglucose Positron Emission Tomography Study in Conscious and Anesthetized Rats. ACS Chem. Nerosci..

[b0175] Lladó-Pelfort L., Assié M.-B., Newman-Tancredi A., Artigas F., Celada P. (2012). In vivo electrophysiological and neurochemical effects of the selective 5-HT1A receptor agonist, F13640, at pre- and postsynaptic 5-HT1A receptors in the rat. Psychopharmacology (Berl).

[b0180] Ma Y., Tang C., Spetsieris P.G., Dhawan V., Eidelberg D. (2007). Abnormal metabolic network activity in Parkinson’s disease: test-retest reproducibility. J. Cereb. Blood Flow Metab. Off. J. Int. Soc. Cereb. Blood Flow Metab..

[b0185] Marcellino D., Kehr J., Agnati L.F., Fuxe K. (2012). Increased affinity of dopamine for D(2) -like versus D(1) -like receptors. Relevance for volume transmission in interpreting PET findings. Synap. N. Y. N.

[b0190] Marsden C.D., Parkes J.D. (1977). Success and problems of long-term levodopa therapy in Parkinson’s disease. Lancet Lond. Engl..

[b0195] McCreary A.C., Varney M.A., Newman-Tancredi A. (2016). The novel 5-HT1A receptor agonist, NLX-112 reduces l-DOPA-induced abnormal involuntary movements in rat: A chronic administration study with microdialysis measurements. Neuropharmacology.

[b0200] Mueller K., Jech R., Ballarini T., Holiga Š., Růžička F., Piecha F.A., Möller H.E., Vymazal J., Růžička E., Schroeter M.L. (2019). Modulatory Effects of Levodopa on Cerebellar Connectivity in Parkinson’s Disease. Cerebellum Lond. Engl..

[b0205] Muñoz A., Li Q., Gardoni F., Marcello E., Qin C., Carlsson T., Kirik D., Di Luca M., Björklund A., Bezard E., Carta M. (2008). Combined 5-HT1A and 5-HT1B receptor agonists for the treatment of L-DOPA-induced dyskinesia. Brain J. Neurol..

[b0210] Muñoz A., Carlsson T., Tronci E., Kirik D., Björklund A., Carta M. (2009). Serotonin neuron-dependent and -independent reduction of dyskinesia by 5-HT1A and 5-HT1B receptor agonists in the rat Parkinson model. Exp. Neurol..

[b0215] Mure H., Hirano S., Tang C.C., Isaias I.U., Antonini A., Ma Y., Dhawan V., Eidelberg D. (2011). Parkinson’s disease tremor-related metabolic network: characterization, progression, and treatment effects. Neuroimage.

[b0220] Newman-Tancredi A., Martel J.-C., Cosi C., Heusler P., Lestienne F., Varney M.A., Cussac D. (2017). Distinctive in vitro signal transduction profile of NLX-112, a potent and efficacious serotonin 5-HT1A receptor agonist. J. Pharm. Pharmacol..

[b0225] Newman-Tancredi A., Depoortère R.Y., Kleven M.S., Kołaczkowski M., Zimmer L. (2021). Translating biased agonists from molecules to medications: Serotonin 5-HT1A receptor functional selectivity for CNS disorders. Pharmacol. Ther..

[b0230] Oh S.W., Shin N.-Y., Yoon U., Sin I., Lee S.-K. (2020). Shared functional neural substrates in Parkinson’s disease and drug-induced parkinsonism: association with dopaminergic depletion. Sci. Rep..

[b0235] Ohno Y., Shimizu S., Tokudome K. (2013). Pathophysiological roles of serotonergic system in regulating extrapyramidal motor functions. Biol. Pharm. Bull..

[b0240] Pagano G., Politis M. (2018). Molecular Imaging of the Serotonergic System in Parkinson’s Disease. Int. Rev. Neurobiol..

[b0245] Paxinos G., Watson C. (2014).

[b0250] Perlbarg V., Lambert J., Butler B., Felfli M., Valabrègue R., Privat A.-L., Lehéricy S., Petiet A., Ariga H. (2018). Alterations of the nigrostriatal pathway in a 6-OHDA rat model of Parkinson’s disease evaluated with multimodal MRI. PLoS One.

[b0255] Poewe W., Seppi K., Tanner C.M., Halliday G.M., Brundin P., Volkmann J., Schrag A.-E., Lang A.E. (2017). Parkinson disease. Nat. Rev. Dis. Primer.

[b0260] Politis M., Wu K., Loane C., Brooks D.J., Kiferle L., Turkheimer F.E., Bain P., Molloy S., Piccini P. (2014). Serotonergic mechanisms responsible for levodopa-induced dyskinesias in Parkinson’s disease patients. J. Clin. Invest..

[b0265] Rylander D., Parent M., O’Sullivan S.S., Dovero S., Lees A.J., Bezard E., Descarries L., Cenci M.A. (2010). Maladaptive plasticity of serotonin axon terminals in levodopa-induced dyskinesia. Ann. Neurol..

[b0270] Schwarting R.K., Huston J.P. (1996). The unilateral 6-hydroxydopamine lesion model in behavioral brain research. Analysis of functional deficits, recovery and treatments. Prog. Neurobiol..

[b0275] Tessitore A., Cirillo M., De Micco R. (2019). Functional Connectivity Signatures of Parkinson’s Disease. J. Park. Dis..

[b0280] Vidal B., Fieux S., Redouté J., Villien M., Bonnefoi F., Le Bars D., Newman-Tancredi A., Costes N., Zimmer L. (2018). In vivo biased agonism at 5-HT1A receptors: characterisation by simultaneous PET/MR imaging. Neuropsychopharmacology.

[b0285] Vidal B., Bolbos R., Redouté J., Langlois J.-B., Costes N., Newman-Tancredi A., Zimmer L. (2020). Pharmacological MRI to investigate the functional selectivity of 5-HT1A receptor biased agonists. Neuropharmacology.

[b0290] Vidal B., Levigoureux E., Chaib S., Bouillot C., Billard T., Newman-Tancredi A., Zimmer L. (2021). Different Alterations of Agonist and Antagonist Binding to 5-HT1A Receptor in a Rat Model of Parkinson’s Disease and Levodopa-Induced Dyskinesia: A microPET Study. J. Park. Dis..

[b0295] Wehrl H.F., Hossain M., Lankes K., Liu C.-C., Bezrukov I., Martirosian P., Schick F., Reischl G., Pichler B.J. (2013). Simultaneous PET-MRI reveals brain function in activated and resting state on metabolic, hemodynamic and multiple temporal scales. Nat. Med..

[b0300] Wichmann T., Delong M.R. (2006). Pathophysiology of Parkinson’s Disease: The MPTP Primate Model of the Human Disorder. Ann. N. Y. Acad. Sci..

