# Supplementary material for: Multimodal imaging study of the 5-HT1A receptor biased agonist, NLX-112, in a model of L-DOPA-induced dyskinesia
Source: Neuroimage Clin. 2023 Aug 15;39:103497. doi: 10.1016/j.nicl.2023.103497 (PMC10474496; doi:10.1016/j.nicl.2023.103497)
Supplement: Supplementary data 1 [file mmc1.docx]

***Supplementary figure 1****: Study design*

***Supplementary figure 2****: Comparison of [^18^F]FDG uptake in the same HPK-LID rat after saline (A) or L-DOPA (B) injection (raw image). These images show an increased radiotracer uptake in the left foreleg (opposite side to the lesioned side) after L-DOPA injection.*

*Left: [^18^F]FDG combined PET/CT, Right : [^18^F]FDG PET*

***Supplementary figure 3****: Comparison of SUV values using [^18^F]FDG PET scans between HPK-non-LID and HPK-LID rats after an acute challenge administration of saline or L-DOPA (6 mg/kg, i.p.).*

*[^18^F]FDG PET mean SUVs in several ROIs after L-DOPA injection in HPK-LID (n=8) and HPK-non-LID rats (n=7). Bars are the mean + SEM; * p < 0.05. Sidak’s multiple comparisons test, following two-way ANOVAs; region factor: F (22, 276) = 0.1303, p>0.9999; group factor: F (22, 276) = 0.5027, p=0.9713; interaction factor: F (1, 276) = 201.2, p<0.0001; L = left (non-lesioned side), R= right (lesioned side). DRN = Dorsal Raphe Nuclei, WB = whole-brain*

***Supplementary figure 4****: Effects of administration of NLX-112 at 0.16 mg/kg on glucose metabolism using [^18^F]FDG PET scans in HPK-non-LID rats following acute i.p. administration of saline or L-DOPA.*

*A - Voxel-to-voxel statistical comparisons of [^18^F]FDG uptake ratio in HPK-non-LID rats between L-DOPA alone and saline injections in HPK-LID rats (on the left; n=7) ; between NLX-112 alone at 0.16 mg/kg and saline injections (on the right; n=7); between co-administration NLX-112 + L-DOPA and saline (bottom; n=7). T scores in color scales (significant increases of glucose metabolism in red and significant decreases in blue; p<0.01, Student’s t test). HPK: hemi-parkinsonian, LID: L-DOPA-induced dyskinesia, NL = non-lesioned side, L = lesioned side. Coronal sections are from +4 to -13 mm with respect to Bregma.*

*B - [^18^F]FDG uptake ratio in several ROIs after L-DOPA, saline injection, NLX-112 alone or co-administration of NLX-112 + L-DOPA in HPK non-LID rats (n=7). Bars are the mean + SEM; * p < 0.05, ** p < 0.01, *** p < 0.001, Tukey’s multiple comparisons test, following two-way ANOVAs. Region factor: F (2.662, 15.97) = 54.49, p<0.0001; treatment factor: F (2.002, 12.01) = 5.838, p=0.0169; interaction factor: F (2.977, 16.83) = 2.939, p=0.0636. L = left (non-lesioned side), R= right (lesioned side), Ctx = cortex*

*C- Functional connectivity analysis between NLX-112 alone and saline in HPK-non-LID rats (n=8) and D- between co-administration of L-DOPA + NLX-112 and saline in HPK-non-LID rats (n=8). Schematic representations (on the left) of the significant changes in functional connectivity (p < 0.001) as measured by comparing the correlation coefficient after Fisher transformation of r values into z values. The dashed arrows represent the significant decreases of functional connectivity between two regions and the solid arrows represent the significant increases of functional connectivity between two regions, as compared to the saline. The correlation matrices (on the right) are expressed as mean values of Pearson correlation coefficient. Positive values of correlation coefficient are shown in red and negative values of correlation coefficient are shown in blue. For statistical comparisons, correlation coefficients were transformed into Z-scores using Fisher transformation before two-way ANOVA followed by Dunnett’s multiple comparisons test (*p < 0.05, ** p < 0.01, **** p < 0.0001).*

***Supplementary figure 5****: Validation of the hemiparkinsonian rat model (HPK) in an additional cohort of n=7 rats (selected using the same criterion defined for the rats used in the imaging experiments, with the apomorphine challenge) using autoradiography with [^11^C]PE2I, a PET radiotracer of dopamine transporters (DAT). All animals show a strong striatal depletion of DAT in the lesioned side compared to the non-lesioned side (mean depletion of -83.52%, p<0.0001, paired t-test). A. Results expressed in optical densities (PSL/mm^2^) and B. Difference between the lesioned side (L) and the non-lesioned side (NL) of binding on a rat brain section.*
